# Test Performance Study on qPCR Assays for Detection of *Phyllosticta citricarpa*

**DOI:** 10.3390/pathogens14050413

**Published:** 2025-04-24

**Authors:** Tjaša Jakomin, Janja Zajc Žunič, Polona Kogovšek

**Affiliations:** 1 Department of Biotechnology and Systems Biology, National Institute of Biology, 1000 Ljubljana, Slovenia; tjasa.jakomin@nib.si; 2 Plant Protection Department, Agricultural Institute of Slovenia, 1000 Ljubljana, Slovenia; janja.zajc@kis.si

**Keywords:** test performance study, *Phyllosticta citricarpa*, real time PCR, *TEF1*

## Abstract

Citrus black spot (CBS), caused by the fungus *Phyllosticta citricarpa*, significantly affects citrus fruit marketability and can lead to premature fruit drop. Accurate and reliable detection of this quarantine pathogen is crucial, particularly for asymptomatic plant material. This study evaluated two qPCR assays, the EPPO recommended assay PC and assay Pc-TEF1, based on *TEF* region, for detecting *P. citricarpa* through a collaborative test performance study (TPS). DNA from the isolates of *Phyllosticta* spp. and other fungi was spiked into citrus fruit peel extracts (lemon, orange, and pomelo) and distributed among 13 laboratories. Sample and qPCR assay stability under typical transport conditions was confirmed, although prolonged storage affected Pc-TEF1 assay performance. The assays were assessed based on sensitivity, specificity, reproducibility, and repeatability. Both assays demonstrated high performance, with repeatability and reproducibility exceeding 95%. The PC assay, as expected, detected different related *Phyllosticta* species, while Pc-TEF1 showed higher specificity for *P. citricarpa* included in the TPS alone. Additionally, inhibitory effects were observed specifically in the pomelo peel samples, suggesting matrix-dependent variability. This TPS confirms that both PC and Pc-TEF1 qPCR assays are robust. Further evaluation of the qPCR assays would support the selection of the most reliable assays for the detection of *P. citricarpa*, contributing to the effective management of CBS disease in citrus production and trade.

## 1. Introduction

The ascomycetous fungus *Phyllosticta citricarpa* (McAlpine) Van der Aa (syn. *Guignardia citricarpa* Kiely) is an economically significant pathogen causing citrus black spot (CBS) disease. CBS significantly reduces the marketability of fruits by inducing symptoms such as hard spots, freckle spots, false melanose, virulent spots on fruits, and lesions on leaves and twigs across nearly all commercially important citrus cultivars [1], particularly lemons (*Citrus limon*) and sweet oranges (*Citrus sinensis*). Severe infections can lead to premature fruit drop [2]. Additionally, asymptomatic infections may occur in fruit and leaf tissues, escaping visual detection [1]. Consequently, the development and application of highly sensitive and reliable diagnostic methods for asymptomatic plant material are crucial.

CBS disease is predominantly found in subtropical humid regions in Africa, Southeast Asia, Australia, and South America. However, in 2019, the occurrence of *P. citricarpa* was confirmed in semi-arid regions of Africa, specifically Tunisia [3], increasing concerns about its potential spread to citrus-growing areas in Europe, particularly given Tunisia’s proximity to the Mediterranean basin. The European Union has established strict regulatory measures for importing and trading citrus fruits and nursery stock, as *P. citricarpa* is classified as a quarantine pathogen in the European Union. Furthermore, Regulation (EU) 2019/1702 [4] mandates annual surveillance for this pathogen in member states. The potential establishment and spread of *P. citricarpa* in EU countries largely depend on climatic conditions, particularly humidity and temperature.

Accurate pathogen detection and identification necessitate diagnostic methods with high sensitivity and specificity. Current diagnostic approaches for *P. citricarpa* include visual inspection for CBS-like symptoms, lesion incubation, isolation, and culturing followed by molecular identification and direct molecular analyses of lesions [5]. Various molecular assays are available [5], among which the quantitative PCR (qPCR) assay developed by Van Gent-Pelzer [6] is most widely employed in diagnostic laboratories (here named PC). Validation data for this assay are available at EPPO [5] and the performance of the assay was demonstrated on different hosts, tissues, and isolates [7,8] and under various laboratory conditions [9]. Novel qPCR assays with improved specificity for distinguishing *P. citricarpa* from its closely related species *P. citriasiana* [8,10] and *P. paracitricarpa* [7,11] have been developed and validated. Two European *P. paracitricarpa* isolates were tested to assess the specificity of the Pc-TEF1 assay targeting the *TEF1* region [11], within which intra-species polymorphisms were later described with comparative genomics, which led to the design of alternative *P. citricarpa* specific assay [7]. A recent phylogenomic study indicated that *P. citricarpa* and *P. paracitricarpa* may be conspecific [12], suggesting that further research is necessary to fully understand the diversity of these fungi and their pathogenicity.

A test performance study was organized in the frame of Euphresco project 2019-A-318: CBS-Detect in 2022 to evaluate two at that time available assays, namely PC [6] and Pc-TEF1 [11], to determine their diagnostic accuracy on three citrus matrixes, i.e., lemon, orange, and pomelo fruit peel. The objective of the study was, thus, to gain data on the repeatability and reproducibility of the assays executed on different equipment, by different chemicals and personnel [13] and supplement the available validation data [9,11].

## 2. Materials and Methods

### 2.1. Sample Collection and Preparation

All the samples were prepared by a test performance study (TPS) organizer. DNA extracted from fungal isolates and other fungi isolates were obtained from the Valencian Institute of Agricultural Research, Spain (IVIA) and Council for Agricultural Research and Economics, Italy (CREA) (Table 1). In total, DNA extracted from three *P. citricarpa* isolates, one *P. citriasiana*, *P. capitalensis*, *P. paracitricarpa*, *Plenodomus tracheiphilus*, and *Colletotrichum* sp. isolates was included in the preparation of the samples. Data on the identity of *Phyllosticta* isolates from culture collections is available in public databases. The identity of isolates IVIA 115 and IVIA 120 was confirmed by the sequencing of ITS and *TEF1* region [14] and sequences were deposited in the database. To mimic samples of asymptomatic fruit, total DNA was extracted from the fruit peels of the following citrus species: lemon (*Citrus limon*), orange (*Citrus sinensis*), and pomelo (*Citrus maxima*), purchased at the local store (Slovenia). DNA was extracted with Nucleospin Plant 2 Kit (Macherey-Nagel, Dueren, Germany) following the previously described protocol [11]. DNA isolated from citrus peel was spiked with known concentration of isolated fungal DNA, mixed, aliquoted, and stored at −20 °C until being dispatched to the participating laboratories. DNA samples and primers and probe (PPS) mixes were sent by courier at ambient temperature. All the samples were coded to ensure full-blind testing of the samples, and coding was random and different for each panel of samples.

### 2.2. Test Selection

Test selection for TPS was based on available qPCR assays and their specificity. At the time of selection, four qPCR assays for *P. citricarpa* with different specificity were published [6,8,11,15]. A diagnostic assay included in the EPPO standard PM7/017(3) [5], referred to herein as PC assay [6], effectively discriminates between *P. citricarpa* from the endophyte *P. capitalensis*. A new assay with higher specificity was developed later, which is capable of discriminating among *P. citricarpa*, *P. citriasiana*, and *P. capitalensis* [8]. Similar discriminatory capabilities are anticipated for the third assay [15], given that its primers and probe target the same genomic region. All three assays target the ITS region which does not allow for discrimination between closely related species and thus, does not offer direct confirmation of the *P. citricarpa*. Despite that, the PC assay was included in the TPS based on the recommendation of the assay in EPPO standard PM7/017(3). Currently, the *TEF1* region is used for barcoding and discrimination between closely related *Phyllosticta* species, e.g., *P. citricarpa* from *P. paracitricarpa* [5], and is targeted with Pc-TEF1 qPCR assay [11]. The development and partial validation of the Pc-TEF1 assay was conducted in the TPS organizing laboratory, leading to its inclusion in the study. Fungi Quant assay (FQ) was used as internal control and detects fungi in general [16] (Table 2).

### 2.3. Real-Time PCR

In preliminary testing, all the reactions were performed in the final volume of 10 μL. 1 × Universal Master Mix (Applied Biosystems, Waltham, MA, USA) was used for PC and Pc-TEF1 assays and Quantabio (Beverly, MA, USA) PerfeCTa^®^ qPCR ToughMix^®^ Low ROX for the FQ assay. Probes and primers were provided by the organizer as a mixture of primers and probes (PPS mix): for Pc-TEF1 and PC assay 300 nM of each primer and 200 nM TaqMan probe were used, and for FQ assay 900 µM and 250 nM of TaqMan probe was used. In preliminary studies, qPCR analyses were performed on a 7900 HT Fast Real-Time PCR System and ViiA 7 Real-Time PCR System (Applied Biosystems) using the cycling conditions 50 °C for 2 min, 95 °C for 10 min, and 45 cycles of 95 °C for 15 s and 65 °C for 1 min, with standard temperature ramping mode (1.6 °C/s).

The real-time PCR data were analyzed using the QuantStudio Real-Time PCR Software v1.3 or SDS v2.4 Software (Applied Biosystems) with automatic baseline. TPS participants were allowed to adjust experimental protocols to the standards of individual laboratories (e.g., use of other reagents than those indicated in the protocol). The participants were asked to record and report all deviations. All the samples were analyzed in triplicate for each of the assays. The assay was considered positive if it produced an exponential amplification curve in at least two out of the three parallel samples tested. The assay was considered negative if it did not produce an amplification curve or if it produced a curve that was not exponential. Cut-off values were set at the Cq value obtained in the negative control (NAC) and values below the round Cq value were considered as positive. Positive amplification controls (PACs) and no template controls (NTCs) were used in each run.

### 2.4. TPS Participants

The criteria for the selection of the participants were defined by the project organizer in accordance with the guidelines for the organization of test performance studies in microbiology [13]. Thirteen laboratories from 11 countries and 3 continents participated in the TPS, and most of the participants were part of the CBS-Detect Euphresco project (2019-A-318) (Table 3). A test performance study was performed according to the EPPO guidelines [17]. The main performance characteristics of assays evaluated within TPS were specificity, reproducibility, and repeatability PM7/98(4) [18]. Repeatability and reproducibility were calculated using the following formula: number of samples where all three repetitions had the same result/total number of samples when the sample was tested under the same or different conditions, respectively. Raw data (Cq values) submitted by the participants were used for the assessment of repeatability and reproducibility and all the signals obtained were considered (no cut-off values were applied). Further, to evaluate the probability of achieving the same test result for identical samples between laboratories, a concordance was calculated [19,20].

### 2.5. Homogeneity and Stability

For homogeneity and stability testing, during the period of TPS, 7 batches of aliquots of all the samples and PPS mixes prepared for TPS were randomly selected and analyzed with assays included in the TPS. The stability testing was carried out under conditions that mimicked transport and storage conditions. This means that the samples were stored at different temperatures and times before testing: (i) up to 23 days at room temperature; (ii) up to 23 days at room temperature + up to 3 weeks at −20 °C.

## 3. Results

### 3.1. Preliminary Evaluation of Samples and Primers and Probe Mixes

**Figure 1:**
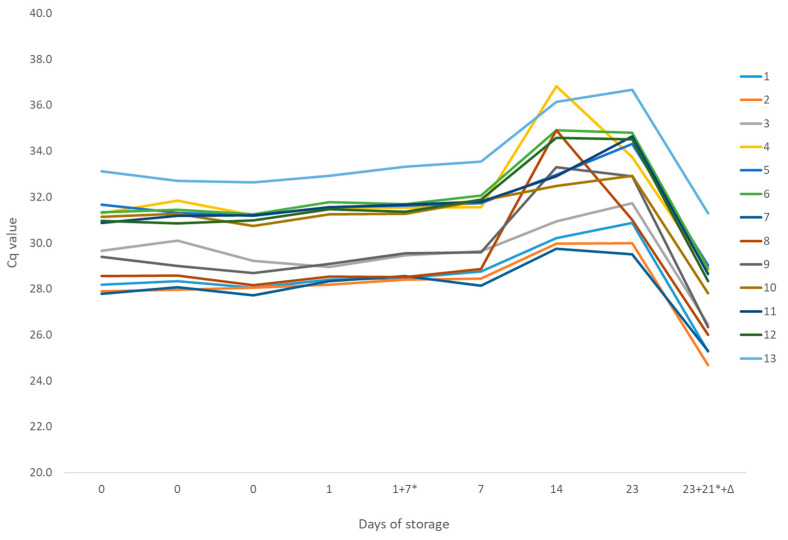
Homogeneity and stability of the samples (1–13, shown with different colors, legend on a graph) shown with average Cq values for the Pc-TEF1 PPS mix. Three sample batches were tested on day 0, and then the stored sample batches were tested on different days after storage at room temperature (1, 7, and 14 days) and at −20 °C (7 days, marked with *). One batch of samples stored for 23 days at room temperature and 21 days at −20 °C was tested with the PPS mix that was stored only at −20 °C (marked with 23 + 21 * + Δ).

The results of the preliminary analysis of the samples and primers and probe (PPS) mixes showed a high homogeneity and stability of the samples, especially with FQ and PC assays, where the coefficient of variation (CV) of the Cq values (calculated from three technical replicates and nine analytical replicates, *n* = 27) was up to 2%. A higher variability was observed in the Pc-TEF1 assay, ranging from 4% to 9%, which was mainly due to analyses performed after 14 days of storage at room temperature. Here, a high variability between technical replicates and an increase in Cq values was observed (Figure 1). However, the variability between the technical replicates and the Cq values decreased when the samples were tested with a PPS mix that was only stored at −20 °C, although the samples were stored at room temperature for 23 days and then at −20 °C for a further 21 days.

### 3.2. Data on Test Performance Study Execution by Participants

The majority of the participants (8/13) received panels of samples and PPS mixes on the same day they were sent, 4 participants received panels the next day, and 1 participant received the panel of samples and PPS mixes 21 days after dispatch. The organizing laboratory also blind-tested a set of samples.

The results obtained by the participants were recorded in an Excel file in which the participants noted the results and any deviations from the recommended protocols. The participants submitted results in the form of Cq values obtained in qPCR runs (raw data) and interpretation of the values as the negative or positive result of testing. The total number of results from a panel of samples tested with one method (data set) was 44: twelve laboratories submitted results for three test methods (FQ, PC, and PC-TEF1), one laboratory submitted results for two methods (FQ and PC), and one laboratory submitted data for two sets of samples (tested on two different instruments). A panel of samples was also blind-tested in the organizing laboratory (laboratory no. 14). Thus, altogether a total of 15 sets of results were included in the calculations for the FQ and the PC assays, and 14 sets of results for the Pc-TEF1 assay.

Seven laboratories used instruments from the same manufacturer as the recommended instruments (i.e., Applied Biosystems), two of the participating laboratories used Qiagen Rotor-Gene (Hilden, Germany) or Roche LightCycler 480 (Basel, Switzerland), and three laboratories used Bio Rad CFX (Hercules, CA, USA) instruments (Supplemental Table S1). One of these laboratories (L-09) used different instruments for the Pc-TEF1 assay (Bio Rad CFX Opus Real-Time PCR Systems) and for the PC and FQ assays (Applied Biosystems StepOne Real-Time PCR System). One laboratory (L-08) tested all three assays on the StepOne Real-Time PCR System and on the CFX96 Touch Real-Time System instrument.

The amplification reagents (reaction mix) used also differed between the laboratories and only two laboratories used the recommended mixes, i.e., Applied Biosystems TaqMan Universal master mix for PC and Pc-TEF-1 assays and QuantaBio for the FQ assay. A total of 10 different mastermix reagents from seven different manufacturers were used (Supplemental Table S1). Most laboratories used the same chemicals for all three assays, with the Applied Biosystems TaqMan Universal master mix being the most frequently used.

One laboratory (L-10) reported problems with the inadequate volume of the reaction mixture for the instrument used for analysis (Qiagen Rotor-Gene Q) and 10 µL of molecular grade water was added to the prepared mixture to obtain the recommended volume (20 µL).

### 3.3. Repeatability and Reproducibility

Repeatability was assessed based on the results of testing an aliquot sample (one biological replicate) in a single laboratory in triplicates for all three assays. At the laboratory level, repeatability ranged from 67% to 100% (Table 4). For assessment of the reproducibility, data interpreted (positive/negative) by the participants were considered. In the case of positive signals in NAC, cut-off values were set (e.g., for L-06) and all the positive signals with exponential curve were considered as valid (e.g., FQ assay in L-11). The reproducibility was high for all three assays, ranging from 73% to 100%, with average reproducibility for each assay ranging from 97% to 98% (Table 5).

### 3.4. Evaluation of Other Assay Performance Criteria

**Figure 2:**
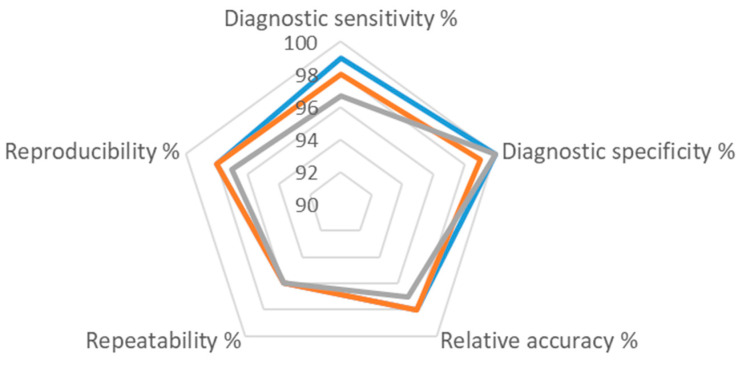
Diagram summarizing the performance of all 3 assays evaluated in the test performance study (FQ assay—gray line; PC assay—blue line; Pc-TEF1 assay—orange line). Numbers present the percentage (%) of each performance characteristic determined (Table 4, Table 5 and Table 6).

Concordance was calculated as the sum of samples that gave the expected results (true positive and true negative) for FQ and PC (*n* = 15) and Pc-TEF1 (*n* = 14) assays in all the laboratories, including both datasets of L-08. The performance of the assays was also evaluated in terms of diagnostic sensitivity and diagnostic specificity by calculating the percentage of results that were true negative (TN), false positive (FP), false negative (FN), true positive (TP), and relatively accurate (Table 6). The interpretation obtained by each laboratory (positive, negative, or inconclusive) was taken into account, with the corrections described above (determination of cut-off values and validity of all the exponential curves). The accuracy of the assays was between 97% and 98%, the diagnostic specificity between 99% and 100%, and the diagnostic sensitivity between 97% and 99% (Figure 2). As it was impossible to interpret the results reported as inconclusive, these were excluded from the analysis.

## 4. Discussion

The performance of two qPCR assays, namely PC [6] and Pc-TEF1 [11], was evaluated for the detection of *P. citricarpa* spp. within a test performance study (TPS) to supplement available validation data. Pc-TEF1 assay was validated during the development of the assay [11] but lacked information on the robustness, i.e., repeatability and reproducibility, of the assay under different testing conditions. Data on the performance of the PC assay was gathered during the evaluation of the assay in various studies [5,7,8]. In addition, a TPS was organized by the Council for Agricultural Research and Economics to evaluate the performance of the PC assay [9], and a new set of *Phyllosticta* target and non-target isolates were spiked into citrus fruit matrixes to supplement the validation data. In parallel a broadly specific fungal control assay, FQ [16], was also assessed.

The specificity of the PS and Pc-TEF1 assays was evaluated on a set of target and non-target *Phyllosticta* species and other citrus-infecting fungi. The PC assay reacted with the isolates of *P. citricarpa* and *P. paracitricarpa* (only recently conspecific [12]) as well as *P. citriasiana* which was expected, as it is based on the highly conserved ITS region. Weak cross-reactivity with *P. citriasiana*, which was observed as high Cq values in some laboratories, was reported also in other studies [5,8,11] but was not detected with *P. citriasiana* isolate ER1891 in the previously organized TPS [9] and could be connected with the host-derived matrix [5]. The Pc-TEF1 assay reacted exclusively with the *P. citricarpa* isolates included in this TPS. However, the observed variability in the *TEF1* region [7] indicates that further evaluation of the specificity of the assay on additional *P. citricarpa* isolates is required. The control FQ assay, intended as a control for fungal DNA extraction efficiency [8], produced positive results across all the tested samples, including the DNA extracted from citrus fruit peel. As the FQ assay detects all fungal DNA [16], even the environmental contamination of the chemical and plastic consumables could yield false positive results. Consequently, laboratory-specific cut-off values were set for laboratories reporting these issues (L-02, L-04, and L-06). Further, reaction mixes could potentially influence the amplification outcomes of the FQ assay, however, no correlation between specific reaction mixes and assay repeatability was identified. Nevertheless, the laboratory-specific verification and determination of appropriate cut-off values remain essential for the FQ assay or any other assay with such broadly reactive assays [21].

The results of the TPS demonstrated high repeatability (>95%) and reproducibility (>96%) for all three assays, supporting their general reliability for *Phyllosticta citricarpa* detection. The PC assay showed even higher repeatability and reproducibility (100%) in the previous TPS [9] and the reasons for lower values in the data analyzed here lie in the inhibitory effect of *C. maxima* fruit peel, giving non-repeatable signals, and individual positive signals with non-target isolates were detected as well. The *P. citriasiana* isolate additionally led to nonrepetitive and nonreproducible results, as the PC assay cross-reacted with this isolate. The cross-reactivity of the PC assay with *P. citriasiana* isolate spiked into *C. maxima* fruit peel DNA was already reported [5], and our data show that pomelo fruit extract affects amplification and detection with FQ assay as well. Further studies are needed to understand and mitigate the mechanisms leading to the inhibition of amplification in qPCR reactions and to fully validate these assays for pomelo fruits. As already reported for the PC assay [7,9], lemon and orange fruit peels did not show any impact on the performance of all three assays evaluated in this TPS.

The homogeneity of the samples and PPS mixes was confirmed by testing three randomly selected sample panels prior to dispatch to participants (Figure 1). The stability of the samples and PPS mixes was assessed under conditions simulating transport, with testing conducted on days corresponding to participants’ receipt of the packages [17]. Stability was confirmed for samples and PPS mixes stored at room temperature for up to 23 days. However, an increase in Cq values and higher standard deviation among technical replicates of the samples tested with Pc-TEF1 assay was noted after 14 days of storage, becoming more pronounced at 23 days. The degradation of target DNA was ruled out by comparison with a PPS mix stored continuously at −20 °C for 21 days and results of the other two assays (PC and FQ), neither of which showed declines in performance. It is likely that components of the Pc-TEF1 PPS mix, particularly the probes’ dye (HEX) and the MGB protein, experienced lower stability and gradual degradation when stored at room temperature, despite protection from light. In general, storage at −20 °C is recommended by the oligo manufacturers and was proven to guarantee longer stability for some qPCR assays [22]. Nevertheless, most participants (8 out of 13) received panels of samples and PPS mixes on the day of dispatch, and an additional 4 received them the next day. One participant received a panel of samples and PPS mixes 21 days after dispatch, and the results aligned with expectations, indicating no significant degradation of the PPS mix or sample DNA during transport and thus validating the reliability of the test outcomes.

Information collected from the participants revealed wide variability among qPCR instruments used across laboratories. While most instruments were suitable for the analysis, the StepOne Real-Time PCR System posed challenges due to its lack of calibration for detecting HEX dye. The participants overcame this issue by either employing alternative instruments or adjusting instrument settings (L-08, L-09). Some participants reported low fluorescence signals from the Pc-TEF1 assay, complicating the differentiation of specific signals from background fluorescence. The use of commonly used reporter dyes that produce strong fluorescence signals would make interpretation of the results more straightforward and less affected by the individual laboratory equipment and internal protocols [23].

Even though the assays were performed reliably across the 14 participating laboratories utilizing 11 distinct qPCR instruments and 10 different reaction mixes, testing of a higher number of samples and *Phyllosticta* isolates would add valuable information on the performance of the assays [10]. The results on the effect of other plant matrixes, like leaves and leaf litter, and hosts (e.g., Citrus *reticulata*) on the performance of the qPCR assays would supplement the available evaluation data and significantly contribute to the general acceptance of the assays. Nevertheless, the diversity and pathology of *P. citricarpa* (with conspecies *P. paracitricarpa*) need to be further explored [12] and corresponding detection methods developed and validated. A comparative study considering all the currently available data, organized across different laboratories, would support the identification of the most robust and accurate testing practices and ease the decision on the method of choice to be introduced in diagnostic laboratories performing routine *P. citricarpa* testing.

## Figures and Tables

**Table 1:** Fungal isolates used in the study. Source or collection of the isolate and origin, year of collection, and original host plant are given where available. The accession number of the ^a^ 28S ribosomal RNA gene, ^b^ whole genome, and ^c^ translation elongation factor 1-alpha (*TEF1*) region are given. Na: no data available.

Fungi	Isolate	Collection/Source	Origin	Collection Year	Original Host Plant	Accession Numbers
*P. citricarpa*	IVIA115	IVIA	Argentina	2014	*C. limon*	PV495870 ^a^, PV527763 ^c^
*P. citricarpa*	IIA-GC003NA	IVIA	Angola	2016	*C. sinensis*	MF693405 ^a^
*P. citricarpa*	CBS 127455	CBS/CREA	Australia	1973	*C. sinensis*	KF206305 ^a^
*P. citriasiana*	IVIA120	IVIA	China	2017	*C. maxima*	PV495871 ^a^, PV527764 ^c^
*P. paracitricarpa*	CBS 141357	CBS/CREA	Greece	2017	*C. limon*	JBBWUC000000000 ^b^
*P. capitalensis*	CBS 120490	CBS/CREA	USA	1968	*C. paradisi*	FJ538338 ^a^, FJ538396 ^c^
*Plenodomus tracheiphilus*	ER 2221	CREA	Italy	Na	*C. sinensis*	Na
*Colletotrichum* sp.	ER 2100	CREA	Italy	Na	*C. maxima*	Na

**Table 2:** PPS mixes, prepared from primers and probes, used in TPS. The final concentrations of the primers and probe in the reaction mix are given.

Assay/PPS Mix		Primer and Probe Sequence	Final Concentration in Reaction	Reference
PC	FP	GGTGATGGAAGGGAGGCCT	0.3 µM	[5,6]
	RP	GCAACATGGTAGATACACAAGGGT	0.3 µM	
	Probe	FAM-AAAAAGCCGCCCGACCTACCTTCA-TAMRA	0.2 µM	
Pc-TEF1	FP	GAAGGTCAGTTGCCTCACACTTT	0.3 µM	[11]
	RP	GTCATATAACCGAGCGCCAAA	0.3 µM	
	Probe	HEX-TTGCGCCTCCACTTG-MGBNFQ	0.2 µM	
FQ	FP	GGRAAACTCACCAGGTCCAG	0.9 µM	[16]
	RP	GSWCTATCCCCAKCACGA	0.9 µM	
	Probe	FAM-TGGTGCATGGCCGTT-MGBNFQ	0.25 µM	

**Table 3:** List of laboratories and country of their origin that participated in the test performance study, listed in alphabetical order.

Participant	Country
Agricultural Institute of Slovenia, Ljubljana	Slovenia
Benaki Phytopathological Institute, Laboratory of Mycology, Kifissia	Greece
Centro Attività Vivaistiche, Faenza	Italy
Centro de Citricultura Sylvio Moreira, Instituto Agronômico, Cordeiropolis, Sao Paulo	Brasil
Council for Agricultural Research and Economics (CREA-DC), Plant Protection and Certification, Rome	Italy
Directorate of plant protection central research institute, Mycology Laboratory, Ankara	Turkey
Fera Science Ltd., York	United Kingdom
French Agency for Food, Environmental and Occupational Health & Safety (ANSES), Plant Health Laboratory, Nancy	France
General directorate of plant Health and input control (DGSVCIA), Quarantine Laboratory, Tunis	Tunisie
Laboratory of Plant Protection Service Lombardy Region, Vertemate con Minoprio	Italy
Main Inspectorate of Plant Health and Seed Inspection, Central Laboratory, Phytosanitary Reference Laboratory, Torun	Poland
National Institute of Biology, Ljubljana	Slovenia
Plant Health Laboratory, DAFM, Celbridge	Ireland
State Plant Protection Service of the Republic of Latvia/The National Phytosanitary Laboratory, Riga	Latvia

**Table 4:** Summary of the repeatability (%) of the assays at laboratory level (L-01 to L-14). L-01 PC assay—for information only, the results were not valid. L-08 provided results obtained on two instruments, the Applied Biosystems instrument (A) and the Bio Rad instrument (B), with the same set of samples. Submitted raw data (Cq values) were included in the calculation of the repeatability.

	L-01	L-02	L-03	L-04	L-05	L-06	L-07	L-08-A	L-08-B	L-09	L-10	L-11	L-12	L-13	L-14
FQ	89%	67%	100%	100%	100%	94%	94%	94%	100%	100%	100%	100%	100%	100%	100%
PC	100%	100%	100%	100%	89%	83%	94%	94%	100%	100%	100%	94%	94%	100%	94%
Pc-TEF1	94%	100%	100%	100%	94%	94%	NA	72%	94%	100%	100%	100%	100%	100%	100%

NA—no results were reported due to technical issues.

**Table 5:** Summary of the reproducibility (%) of the FQ, PC, and Pc-TEF1 assays at sample level (1–18) and average reproducibility at the assay level. Sixteen samples were prepared from the DNA extracted from different fungal isolates, and spiked into the DNA extracted from citrus fruit peel (host background DNA): one sample contained fungal DNA only (no. 13), and one sample consisted of citrus peel DNA only (no. 18).

Sample No.	Isolate	Host Background DNA	Fungi	FQ	PC	Pc-TEF1
1	IVIA115	*Citrus limon*	*P. citricarpa*	100%	100%	100%
2	IVIA115	*Citrus sinensis*	*P. citricarpa*	100%	100%	100%
3	IVIA115	*Citrus maxima*	*P. citricarpa*	93%	100%	100%
4	IVIA115	*C. limon*	*P. citricarpa*	100%	100%	93%
5	IVIA115	*C. sinensis*	*P. citricarpa*	100%	100%	100%
6	IVIA115	*C. maxima*	*P. citricarpa*	93%	100%	100%
7	IIA-GC003NA	*C. limon*	*P. citricarpa*	100%	100%	100%
8	IIA-GC003NA	*C. sinensis*	*P. citricarpa*	100%	100%	100%
9	IIA-GC003NA	*C. maxima*	*P. citricarpa*	93%	100%	100%
10	IIA-GC003NA	*C. limon*	*P. citricarpa*	100%	100%	93%
11	IIA-GC003NA	*C. sinensis*	*P. citricarpa*	100%	100%	100%
12	IIA-GC003NA	*C. maxima*	*P. citricarpa*	100%	100%	93%
13	CBS 127455	*/*	*P. citricarpa*	100%	100%	100%
14	IVIA120	*C. maxima*	*P. citriasiana*	73%	73%	93%
15	CBS141357	*C. sinensis*	*P. paracitricarpa*	100%	100%	100%
16	CBS 120490	*C. limon*	*P. capitalensis*	100%	93%	100%
17	ER2221 + ER2100	*C. limon*	*Plenodomus tracheiphilus + Colletotrichum* sp.	100%	93%	100%
18	*C. limon + C. sinensis + C. maxima*		/	87%	100%	100%
Average reproducibility				97%	98%	98%

**Table 6:** Comparison of the performance parameters determined for the individual assay across all the submitted data sets. Legend: INC = inconclusive; TN = true negative; FP = false positive; FN = false negative; TP = true positive; % = percentage. For the calculation of the diagnostic parameters from the diagnostic sensitivity to the diagnostic odds ratio, the inconclusive results were excluded. Na—not applicable (all samples were positive).

Diagnostic Parameter	FQ	PC	Pc-TEF1
Total data sets	15	15	14
Expected positives	270	225	182
Total data points	270	270	252
INC	0	3	0
TN	0	43	69
FP	0	0	1
FN	9	3	3
TP	261	221	179
INC%	0	1	0
TN%	0	16	27
FP%	0	0	0.4
FN%	3	1	1
TP%	97	82	71
Concordant	261	264	248
Non-concordant	9	6	4
Concordant%	97	98	98
Non-concordant%	3	2	2
Diagnostic sensitivity%	97	99	98
Diagnostic specificity%	na	100	99
False positive rate%	na	0	1
False negative rate%	3	1	2
Relative accuracy%	97	98	98
Power%	97	98	98
Positive predictive value%	100	100	99
Negative predictive value%	na	96	96
Diagnostic odds ratio	na	na	4117

## Data Availability

The original contributions presented in this study are included in the article/Supplementary Materials. Further inquiries can be directed to the corresponding author.

## Supplementary Materials

The following supporting information can be downloaded at https://www.mdpi.com/article/10.3390/pathogens14050413/s1, Table S1: Real-time PCR instruments and reaction mixes used for the analysis of the samples; Table S2: Raw qPCR data provided by participants.

## References

[1] Parnell S., Schenk M., Schrader G., Vicent A., Delbianco A., Vos S. (2020). Pest Survey Card on *Phyllosticta citricarpa*. EFSA Support. Publ..

[2] Kotzé J.M. (1981). Epidemiology and Control of Citrus Black Spot in South Africa. Plant Dis..

[3] Boughalleb-M’Hamdi N., Fathallah A., Benfradj N., Mahmoud S.B., Ali A.B.H., Medhioub L., Jaouadi I., Huber J., Jeandel C., Ioos R. (2020). First Report of Citrus Black Spot Disease Caused by *Phyllosticta citricarpa* on *Citrus limon* and *C. sinensis* in Tunisia. New Dis. Rep..

[4] REGULATION (EU) 2019/1702; European Commission Delegated Regulation (EU) 2019/1702 of 1 August 2019 Supplementing Regulation (EU) 2016/2031 of the European Parliament and of the Council by Establishing the List of Priority Pests. *Off. J. Eur. Union*, Brussels, Bulgium, 2019. http://data.europa.eu/eli/reg_del/2019/1702/oj/eng.

[5] EPPO (2020). PM 7/017 (3) *Phyllosticta citricarpa* (Formerly *Guignardia citricarpa*). EPPO Bull..

[6] Van Gent-Pelzer M.P.E., Van Brouwershaven I.R., Kox L.F.F., Bonants P.J.M. (2007). A TaqMan PCR Method for Routine Diagnosis of the Quarantine Fungus *Guignardia citricarpa* on Citrus Fruit. J. Phytopathol..

[7] Ioos R., Puertolas A., Renault C., Ndiaye A., Cerf-Wendling I., Hubert J., Wang W., Jiao C., Li H., Armengol J. (2023). Harnessing the Power of Comparative Genomics to Support the Distinction of Sister Species within *Phyllosticta* and Development of Highly Specific Detection of *Phyllosticta citricarpa* Causing Citrus Black Spot by Real-Time PCR. PeerJ.

[8] Schirmacher A.M., Tomlinson J.A., Barnes A.V., Barton V.C. (2019). Species-specific Real-time PCR for Diagnosis of *Phyllosticta citricarpa* on *Citrus* Species. EPPO Bull..

[9] CREA Validation Process of the Real Time PCR for the Identification of *Phyllosticta citricarpa* (van Gent-Pelzer et al., 2007). https://dc.eppo.int/validation_data/dwvalidation?id=140.

[10] Ahmed Y., Hussein A., Hubert J., Fourrier-Jeandel C., Aguayo J., Ioos R. (2020). New Multiplex Conventional PCR and Quadruplex Real-Time PCR Assays for One-Tube Detection of *Phyllosticta citricarpa*, *Elsinoë fawcettii*, *Elsinoë australis*, and *Pseudocercospora angolensis* in *Citrus*: Development and Validation. Appl. Microbiol. Biotechnol..

[11] Zajc J., Kogej Z., Fišer S., Gostinčar C., Vicent A., Galvañ Domenech A., Ricconi L., Boonham N., Ravnikar M., Kogovšek P. (2023). Highly Specific qPCR and Amplicon Sequencing Method for Detection of Quarantine Citrus Pathogen *Phyllosticta citricarpa* Applicable for Air Samples. Plant Pathol..

[12] van Ingen-Buijs V.A., van Westerhoven A.C., Skiadas P., Zuijdgeest X.C.L., Haridas S., Daum C., Duffy K., Guo J., Hundley H., LaButti K. (2024). *Phyllosticta paracitricarpa* Is Synonymous with the EU Quarantine Fungus *P. citricarpa* Based on Phylogenomic Analyses. Fungal Genet. Biol..

[13] Vučurović A., Mehle N., Anthoine G., Dreo T., Ravnikar M. (2022). Critical Points for the Organisation of Test Performance Studies in Microbiology: Plant Pathogens as a Case Study.

[14] EPPO (2021). PM 7/129 (2) DNA Barcoding as an Identification Tool for a Number of Regulated Pests. EPPO Bull..

[15] Hu J., Johnson E.G., Wang N.-Y.Y., Davoglio T., Dewdney M.M. (2014). qPCR Quantification of Pathogenic *Guignardia citricarpa* and Nonpathogenic *G. mangiferae* in Citrus. Plant Dis..

[16] Liu C.M., Kachur S., Dwan M.G., Abraham A.G., Aziz M., Hsueh P.R., Huang Y.T., Busch J.D., Lamit L.J., Gehring C.A. (2012). FungiQuant: A Broad-Coverage Fungal Quantitative Real-Time PCR Assay. BMC Microbiol..

[17] EPPO (2022). PM 7/122 (2) Guidelines for the Organization of Interlaboratory Comparisons by Plant Pest Diagnostic Laboratories. EPPO Bull..

[18] EPPO (2019). PM 7/98 (4) Specific Requirements for Laboratories Preparing Accreditation for a Plant Pest Diagnostic Activity. EPPO Bull..

[19] Langton S.D., Chevennement R., Nagelkerke N., Lombard B. (2002). Analysing Collaborative Trials for Qualitative Microbiological Methods: Accordance and Concordance. Int. J. Food Microbiol..

[20] Massart S., Lebas B., Chabirand A., Chappé A.-M., Dreo T., Faggioli F., Harrison C., Macarthur R., Mehle N., Mezzalama M. (2022). Guidelines for Improving Statistical Analyses of Validation Datasets for Plant Pest Diagnostic Tests. EPPO Bull..

[21] Ioos R., Fourrier C., Iancu G., Gordon T.R. (2009). Sensitive Detection of *Fusarium circinatum* in Pine Seed by Combining an Enrichment Procedure with a Real-Time Polymerase Chain Reaction Using Dual-Labeled Probe Chemistry. Phytopathology.

[22] Lopez M.L.D., Crichton E.M., Allison M.J., Dema A.H., Bonderud M.T., Helbing C.C. (2024). Effects of Storage Conditions on the Stability of qPCR Reagents: Implications for Environmental DNA Detection. BMC Res. Notes.

[23] Ioos R., Aloi F., Piškur B., Guinet C., Mullett M., Berbegal M., Bragança H., Cacciola S.O., Oskay F., Cornejo C. (2019). Transferability of PCR-Based Diagnostic Protocols: An International Collaborative Case Study Assessing Protocols Targeting the Quarantine Pine Pathogen *Fusarium circinatum*. Sci. Rep..

